# Supplementary material for: Genetic subtypes of smoldering multiple myeloma are associated with distinct pathogenic phenotypes and clinical outcomes
Source: Nat Commun. 2022 Jun 15;13:3449. doi: 10.1038/s41467-022-30694-w (PMC9200804; doi:10.1038/s41467-022-30694-w)

Supplementary Information:

Genetic Subtypes of Smoldering Multiple Myeloma are associated with Distinct Pathogenic Phenotypes and Clinical Outcomes

Tables and Figures with legends:

Supplementary Table 1A: Baseline demographics and clinical characteristics of the SMM patients in the primary cohort (n=214). Numerical Variables are described as medians and the range of values, while categorical variables are presented as numbers and percentages.

	**Total** n = 214 (%)
**Age**	
Median (range)	62 (34 - 85)
**Sex**	
Female	115 (54)
Male	99 (46)
**Race**	
Black or African American	10 (5)
White	204 (95)
**BM % involvement**	
Median (range)	0.250 (0.028 - 0.800)
**M-spike**	
Median (range)	1.80 (0.00 - 5.17)
**LDH**	
Median (range)	174.5 (92.0 - 562.0)
Missing	*24 (11)*
**β2M**	
Median (range)	2.40 (0.80 - 9.10)
**Mayo, 2008**	
Low	48 (22)
Intermediate	136 (64)
High	30 (14)
**20-2-20 Risk score**	
Low	53(25)
Intermediate	63(29)
High	98(46)

Supplementary Table 1B: Significant genetic features in the six identified clusters that were positively associated with each cluster in the primary cohort (n=214). The features were determined by a one-sided Fisher test and ranked by significance (Benjamini-Hochberg adjusted p value < 0.1).

Cluster	Genetic feature	P value	Adjusted P value
**HL1**	NRAS	3.23E-05	1.58E-03
	TRAF3	8.11E-05	1.99E-03
	MAX	1.27E-03	1.55E-02
	FAM46C	8.40E-03	8.23E-02
**HL2**	16q_del	6.26E-09	3.07E-07
	t(14;20)	6.60E-07	1.62E-05
	6q_del	1.87E-06	3.05E-05
	MAFB	1.19E-05	1.46E-04
	BRAF	3.12E-04	3.06E-03
	del_20q	7.52E-04	6.14E-03
	del_18q	1.27E-03	8.88E-03
	NRAS	1.64E-03	1.00E-02
	DUSP2	3.71E-03	1.82E-02
	1p_del	5.19E-03	2.12E-02
	4q_del	4.47E-03	1.99E-02
	TP53	8.40E-03	2.74E-02
	ATM	8.40E-03	2.74E-02
	NFKB2	8.40E-03	2.74E-02
	17p_del	1.46E-02	4.46E-02
	LTB	1.62E-02	4.67E-02
**HL3**	KRAS	6.38E-07	3.13E-05
	NFKBIA	3.71E-03	6.06E-02
	MYC translocation	3.71E-03	6.06E-02
**HL4**	2p_amp	2.36E-06	1.16E-04
	NFKB2	3.13E-04	7.67E-03

Cluster	Genetic feature	P value	Adjusted P value
**TL1**	t(14;16)	6.29E-08	2.87E-06
	t(4;14)	1.17E-07	2.87E-06
	14q_del	1.87E-06	3.05E-05
	DIS3	1.10E-04	7.54E-04
	MAF	8.11E-05	7.54E-04
	17p_del	1.39E-04	7.54E-04
	12p_del	1.10E-04	7.54E-04
	8p_del	1.33E-04	7.54E-04
	10p_del	1.10E-04	7.54E-04
	HIST1H1E	9.01E-04	4.01E-03
	ZNF292	1.27E-03	4.78E-03
	SETD2	1.27E-03	4.78E-03
	PRKD2	3.71E-03	1.07E-02
	22q_del	3.31E-03	1.07E-02
	9q_amp	3.71E-03	1.07E-02
	ATM	8.40E-03	2.06E-02
	EGR1	8.40E-03	2.06E-02
**TL2**	t(11;14)	1.54E-19	7.55E-18
	11q_amp	3.12E-04	7.65E-03

Supplementary Table 2: Baseline demographics and clinical characteristics of the 87 untreated SMM patients. These patients were followed for disease progression and their clinical stratification was according to the 20/2/20 clinical risk model. Variables in each group are described as medians with the range of values. Comparison between the different risk groups is done using Cuzick's trend test.

	**Total** n = 87 (%)	**20-2-20 clinical model** Low n = 23 (27)	Intermediate n = 22 (26)	High n = 42 (55)	***p-value***
**BM % involvement**					
Median (range)	0.30 (0.10 - 0.80)	0.15 (0.10 - 0.20)	0.20 (0.10 - 0.55)	0.40 (0.20 - 0.80)	*< 0.001*†
**M-spike**					
Median (range)	1.69 (0.00 - 5.17)	1.18 (0.00 - 1.87)	1.34 (0.00 - 2.30)	2.16 (0.00 - 5.17)	*< 0.001*†
**FLCr, Involved/uninvolved ratio**					
Median (range)	19.3 (1.1 - 325.3)	2.9 (1.1 - 15.7)	11.1 (1.6 - 255.9)	48.6 (2.3 - 325.3)	*< 0.001*†
**β2M**					
Median (range)	2.50 (1.40 - 6.40)	2.50 (1.50 - 6.40)	2.35 (1.50 - 3.80)	2.50 (1.40 - 5.90)	*0.95*†
**Hemoglobin**					
Median (range)	12.6 (8.3 - 16.2)	12.7 (8.3 - 15.4)	13.2 (11.0 - 16.2)	12.4 (10.3 - 15.2)	*0.14*†
**LDH**					
Median (range)	156 (92 - 422)	147 (92 - 422)	172 (120 - 262)	160 (104 - 364)	*0.21*†
Missing	*15 (18)*	*6 (26)*	*7 (32)*	*2 (5)*	
**Calcium**					
Median (range)	9.60 (8.70 - 10.50)	9.60 (8.70 - 10.50)	9.45 (8.80 - 10.00)	9.60 (9.00 - 10.10)	*0.82*†
**Creatinine**					
Median (range)	0.90 (0.60 - 1.60)	0.90 (0.69 - 1.40)	0.99 (0.60 - 1.60)	0.90 (0.60 - 1.40)	*0.12*†
Missing	*1 (1)*	-	*1 (5)*	-	
**Total protein**					
Median (range)	8.1 (5.5 - 11.0)	7.7 (6.8 - 8.4)	7.8 (5.5 - 8.8)	8.6 (5.5 - 11.0)	*< 0.001*†
**Albumin**					
Median (range)	4.1 (2.9 - 5.0)	4.2 (3.5 - 5.0)	4.2 (3.5 - 4.8)	4.0 (2.9 - 4.8)	*0.10*†
Missing	*1 (1)*	*1 (4)*	-	-	
**Globulin**					
Median (range)	3.8 (0.3 - 7.8)	3.7 (2.2 - 4.6)	3.5 (2.0 - 4.6)	4.2 (0.3 - 7.8)	*0.003*†
Missing	*1 (1)*	*1 (4)*	-	-	
**Albumin/Globulin ratio**					
Median (range)	1.1 (0.4 - 13.0)	1.1 (0.8 - 2.3)	1.2 (0.9 - 1.8)	0.9 (0.4 - 13.0)	*0.005*†
Missing	*1 (1)*	*1 (4)*	-	-	

Supplementary Table 3: List of genetic alterations that were significantly enriched in High-risk genetic subgroups (HL2, TL1, HL3) in the primary cohort (n=214). The features were determined by a two-sided Fisher test and ranked by significance (Benjamini-Hochberg adjusted p value < 0.1).

Genetic alteration	P value	Adjusted P value
16q_del	3.92133701244461E-19	1.88224176597341E-17
6q_del	1.30661737533785E-16	3.13588170081083E-15
del_22q	3.46052641707861E-15	5.53684226732578E-14
del_20q	9.81983940020603E-14	1.17838072802472E-12
KRAS	5.74309437412292E-12	5.09021145715801E-11
1p_del	6.36276432144751E-12	5.09021145715801E-11
del_8p	1.076934966262E-11	7.38469691151089E-11
17p_del	2.66210661752379E-10	1.59726397051427E-09
t(4;14)	9.59521520668869E-10	5.11744811023397E-09
14q_del	7.16829126182379E-09	3.44077980567542E-08
del_4q	1.03923060318069E-08	4.534824450243E-08
ATM	6.28534662885084E-08	2.32074337065262E-07
t(14;16)	6.28534662885084E-08	2.32074337065262E-07
DIS3	7.72727909076587E-08	2.64935283111973E-07
BRAF	5.07514260624449E-07	1.52254278187335E-06
LTB	6.5996139602957E-07	1.6672708952326E-06
HIST1H1E	6.5996139602957E-07	1.6672708952326E-06
t(14;20)	6.5996139602957E-07	1.6672708952326E-06
NFKBIA	7.64717173177041E-07	1.8353212156249E-06
del_10p	3.75087303233623E-06	8.18372297964268E-06
TP53	6.63104069343861E-06	1.32620813868772E-05
ZNF292	1.19077388394712E-05	1.97093608377455E-05
MAFB	1.19077388394712E-05	1.97093608377455E-05
1q_gain	3.91221254141483E-05	6.25954006626372E-05
FAM46C	4.98897812477866E-05	7.72486935449599E-05
MAF	8.10600203567709E-05	0.000111168027917857

Supplementary Table 4: Performance of the Clinical Models only and with adding the Genetic Model (based on the genetic risk groups). Improvement in goodness of fit was assessed with a likelihood ratio test. The genetic model significantly improved the fit of the clinical-only models. A global assessment of each model was also assessed using a C-statistic for censored survival data. The statistic for each time-to-event model is reported with a 95% CI. Values range from 0.5 to 1, which indicates a poor to perfect model.

**Cohort**	**Model**	**Likelihood Ratio Test Statistic**	**χ2 *P***	**Model C-statistic (95% CI)**	**AIC/BIC**
Primary (DFCI, UCL, Greece) (n=87)	Clinical	14.7	< 0.001	0.71 (0.64 - 0.78)	406/411
	Clinical + Genetic			0.76 (0.68 - 0.83)	396/404
Validation (UAMS) (n=75)	Clinical	12.1	0.002	0.65 (0.49 - 0.80)	165/168
	Clinical + Genetic			0.76 (0.62 - 0.90)	157/162
Validation (UAMS, Mayo) (n=142)	Clinical	13.53	0.001	0.66 (0.59 - 0.74)	567/571
	Clinical + Genetic			0.71 (0.64 - 0.78)	560/569
Combined (Primary, UAMS, Mayo) (n=229)	Clinical	29.84	< 0.001	0.69 (0.64 - 0.73)	1146/1151
	Clinical + Genetic			0.74 (0.69 - 0.78)	1123/1135

Supplementary Table 5: Univariate Cox PFS regression of the high-risk genetic features in the primary cohort (n = 87). Number of patients with event and percentages from the total number of patients evaluable, HR hazards ratio, error bars indicate 95% CI. All p values are two-sided. Naïve and corrected p-values are presented. Multiple testing correction was done by Benjamini-Hochberg method with p value < 0.1.

		**Estimates**		**p-value**	
**Variable**	**No. of patients (%)**	**HR**	**95% CI**	**naive**	**corrected**
MYC alterations	7 (9)	7.58	3.02 - 19.03	< 0.001	< 0.001
KRAS	12 (14)	4.13	2.10 - 8.12	< 0.001	< 0.001
del(1p)	7 (8)	4.24	1.89 - 9.51	< 0.001	0.003
del(14q)	10 (12)	3.06	1.47 - 6.34	0.003	0.010
del(8p)	8 (9)	3.43	1.53 - 7.70	0.003	0.010
TP53	5 (6)	3.89	1.50 - 10.10	0.005	0.017
NRAS	5 (6)	3.23	1.27 - 8.21	0.014	0.035
del(6q)	11 (13)	2.46	1.18 - 5.11	0.016	0.035
del(22q)	7 (8)	2.97	1.24 - 7.09	0.014	0.035
t(4;14)	5 (6)	2.69	1.05 - 6.89	0.039	0.072
del(16q)	18 (21)	1.66	0.89 - 3.08	0.11	0.06
del(4q)	5 (6)	1.69	0.61 - 4.69	0.32	0.41
del(13q)	42 (49)	1.26	0.73 - 2.17	0.41	0.50
gain(1q)	21 (25)	1.6	0.90 - 2.83	0.47	0.12

Supplementary Table 6: Univariate Cox PFS regression of the high-risk genetic features in the combined cohorts (n = 229). Number of patients with event and percentages from the total number of patients evaluable, HR hazards ratio, error bars indicate 95% CI. All p values are two-sided. Naïve and corrected p-values are presented. Multiple testing correction was done by Benjamini-Hochberg method with p value < 0.1.

		Estimates	p-value	
**Variable**	**No. of patients (%)**	**HR (95% CI)**	**naive**	**corrected**
MYC aberrations	48 (21)	1.9 (1.2-2.8)	0.0022	0.0055
KRAS	43 (19)	2.7 (1.9-4)	3.50E-07	<0.0001
gain_1q	60 (26)	1.5 (1-2.1)	0.047	0.0588
del_16q	38 (17)	1.4 (0.91-2.2)	0.12	0.1385
del_1p	24 (11)	2 (1.3-3.3)	0.0027	0.0058
del_8p	20 (9)	3.2 (1.9-5.4)	1.10E-05	0.0001
del_6q	29 (13)	2.6 (1.7-4.1)	2.10E-05	0.0001
t.4.14.	16 (7)	2.6 (1.5-4.6)	0.00082	0.0025
TP53_aberrations	14 (6)	2.6 (1.4-4.8)	0.0035	0.0066
DIS3	13 (6)	2.3 (1.2-4.3)	0.0082	0.0123
FAM46C	9 (4)	2.7 (1.3-5.7)	0.0067	0.0112
del_22q	22 (10)	1.7 (1-2.9)	0.039	0.0532
del_14q	23 (10)	2.6 (1.6-4.3)	0.00011	0.0004
t.14.16.	6 (3)	0.98 (0.35-2.7)	0.97	0.9700
ATM	6 (3)	2.1 (0.75-5.6)	0.16	0.1714

Supplementary Figure 1: Outline and results of the binary matrix factorization and consensus clustering. A) Age and sex distribution of primary cohort (n=214). B) Logistic principal components analysis (PCA) projection of binarized dataset (n=214). Green color denotes the presence of the variable of interest while red color indicates the rest of the dataset. C) Sample-sample consensus matrix of binary matrix factorization results (K=2-10) with translocations and copy number (left). D) LogisticPCA colored by subtypes. Mutational signature abundance per molecular subtype for E) Aging , F) APOBEC, and G) AID signatures. H) APOBEC mutational signature abundance in HL2/TL1 vs the other clusters. Two-sided p-value was calculated using the Wilcoxon rank-sum test. Boxplots representing median, and interquartile range, whiskers representing first, and fourth quartile

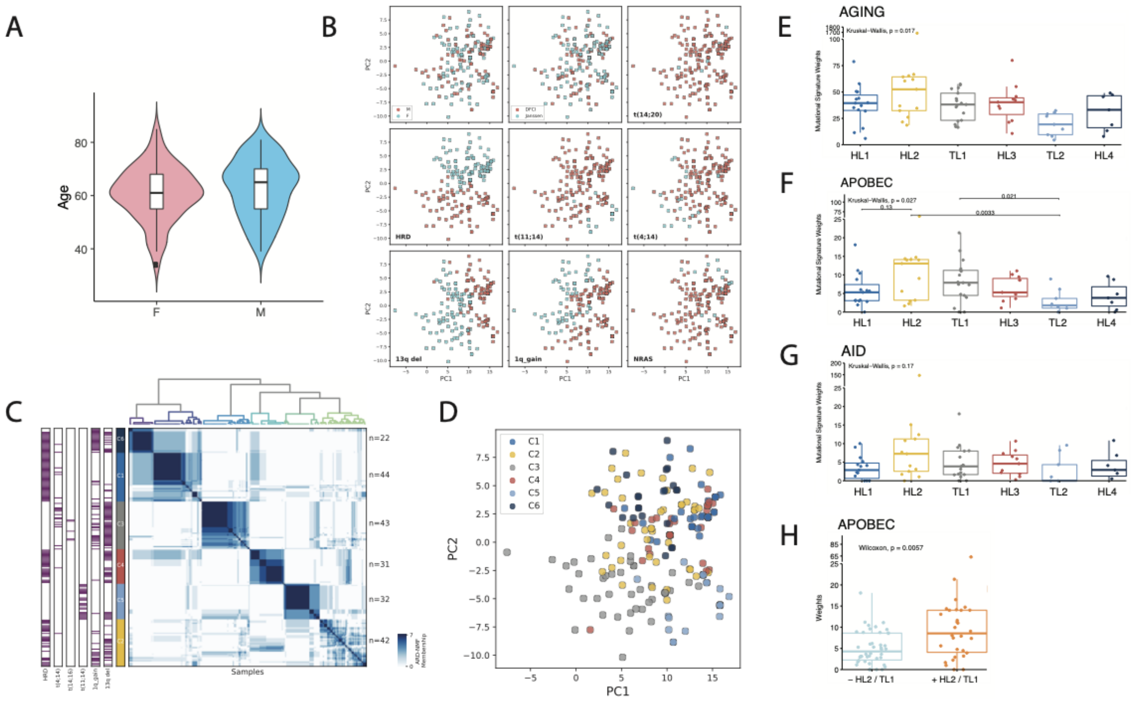

Supplementary Figure 2: Additional gene expression (log TPM + 1) comparisons (n=89). A) *CCND1* gene in TL2 tumors vs. the other tumors; B) *CCND2* gene in TL1 & HL2 tumors vs. the rest. C) *CCND2* expression in HL1-4 (Hyperdiploid) subtypes with and without 11q gain. D) *CCND1* expression in HL1-4 subtypes with and without 11q gain. Two-sided p-value was calculated using the Wilcoxon rank-sum test. Expression is measured by the log2 value of transcript per million of each gene (log2 TPM +1). Boxplots representing median, and interquartile range, whiskers representing first, and fourth quartile. Two-sided p-value was calculated using the Wilcoxon rank-sum test.

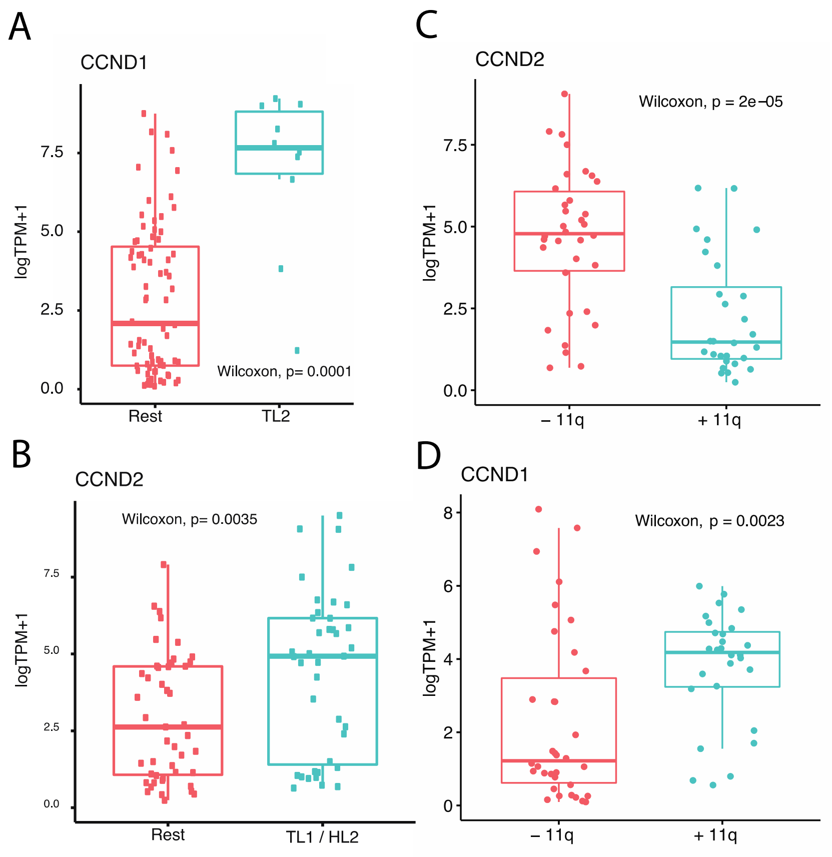

Supplementary Figure 3:

A) Kaplan-Meier curves for analysis of TTP according to the six genetic subtypes from the primary cohort. **B)** Cox proportional hazards analysis of the six genetic subtypes and in the primary cohort**. C)** Kaplan-Meier curves for analysis of TTP in patients from the primary cohort belonging to high vs intermediate and low-risk genetic subtypes in the clinically high-risk group by the 20-2-20 model. All p values are two-sided. Differences in survival curves and subsequent two-sided p values were calculated using the log-rank test. Forest plots are used to visualize the multivariate analysis. N: number of patients with event and percentages from the total number of patients evaluable, HR hazards ratio, error bars indicate 95% CI.

A)

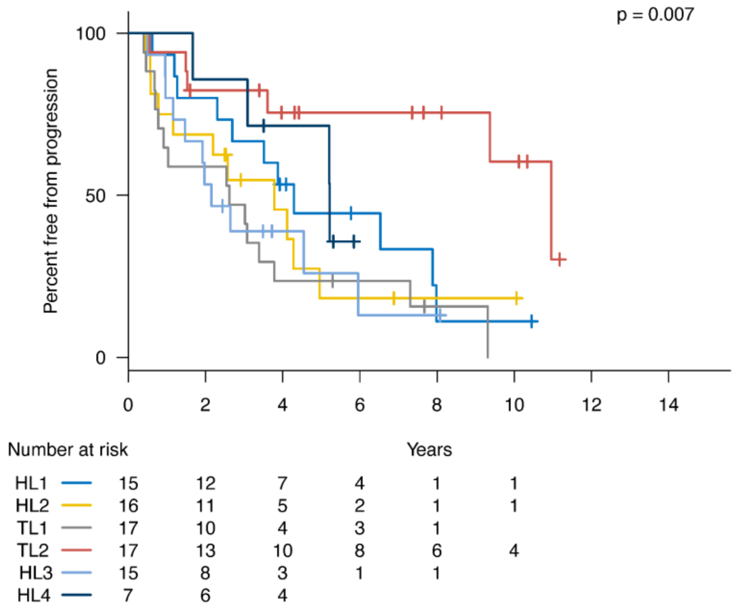

B)

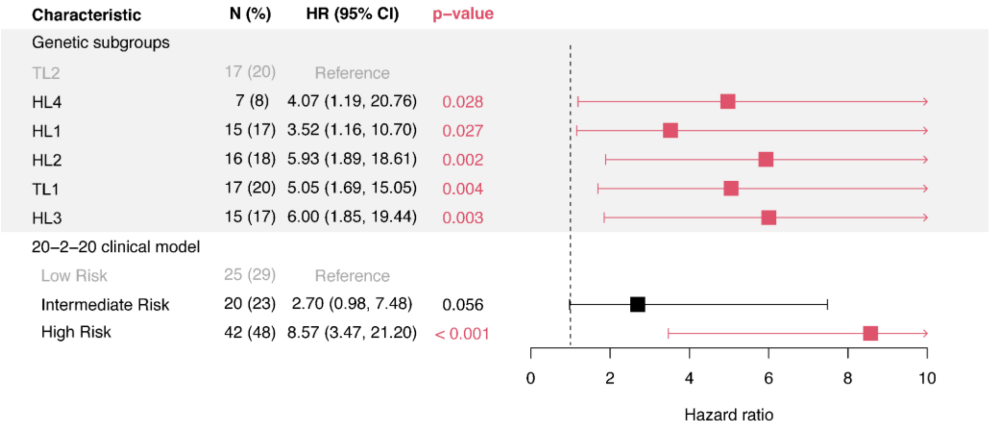
Characteristic
N (%)
HR (95% CI)
p–value
Genetic subgroups
TL2 17 (20) Reference
HL4 7 (8) 4.07 (1.19, 20.76) 0.028
HL1 15 (17) 3.52 (1.16, 10.70) 0.027
HL2 16 (18) 5.93 (1.89, 18.61) 0.002
TL1 17 (20) 5.05 (1.69, 15.05) 0.004
HL3 15 (17) 6.00 (1.85, 19.44) 0.003
20–2–20 clinical model
Low Risk 25 (29) Reference
Intermediate Risk 20 (23) 2.70 (0.98, 7.48) 0.056
High Risk 42 (48) 8.57 (3.47, 21.20) < 0.001
0
2
4
6
8
10
Hazard ratio

C)

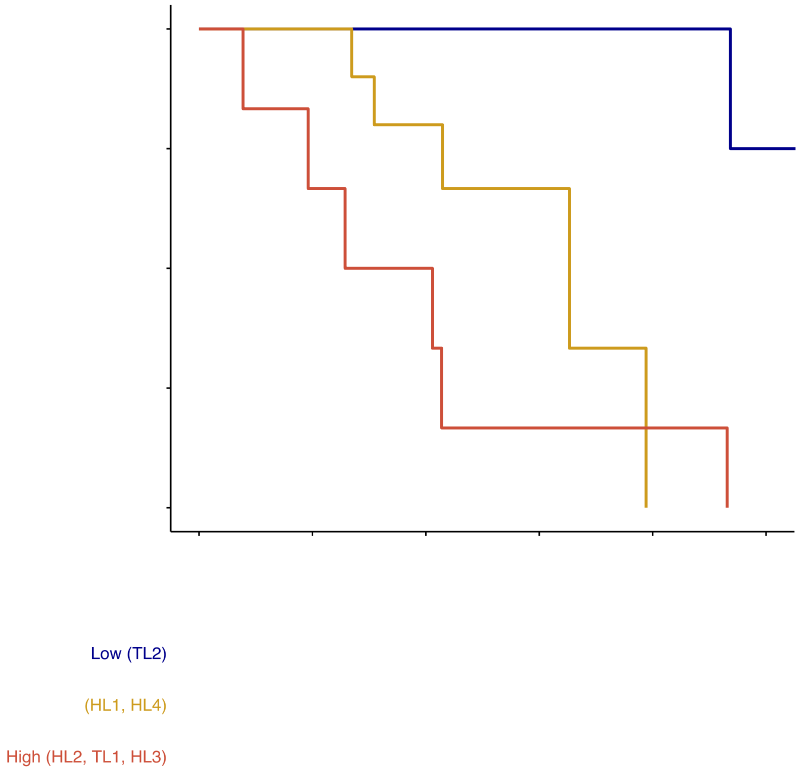
Low (TL2)
(HL1, HL4)
High (HL2, TL1, HL3)

Supplementary Figure 4: A) Random Forest K-fold cross validation accuracy over 10 repeats with mean accuracy and standard deviation reported. **B)** Grid search to optimize parameters did not improve over initial random forest parameters. **C)** The prevalence of each feature in the dataset plotted by feature importance in classifying genetic subtypes.

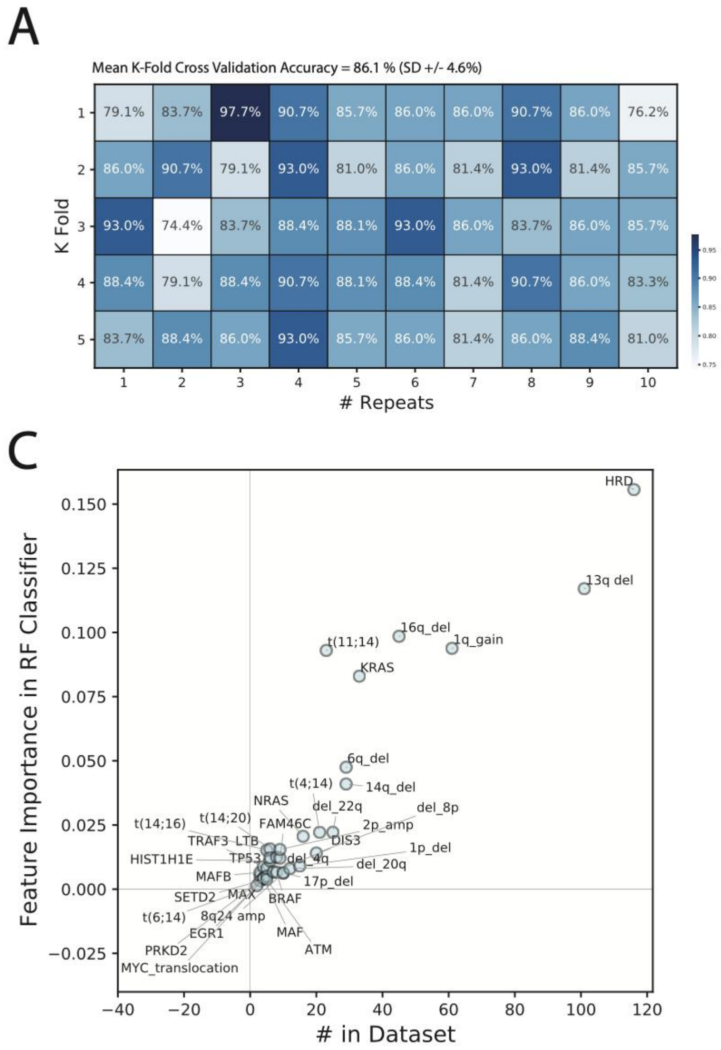

Supplementary Figure 5: Kaplan-Meier curves for analysis of TTP in the combined cohort (n=229) belonging to high vs intermediate and low-risk genetic subtypes in the clinically high(A) and intermediate(B)groups by the 20-2-20 model in the combined cohort. All p values are two-sided. Differences in survival curves and subsequent two-sided p values were calculated using the log-rank test.

A)

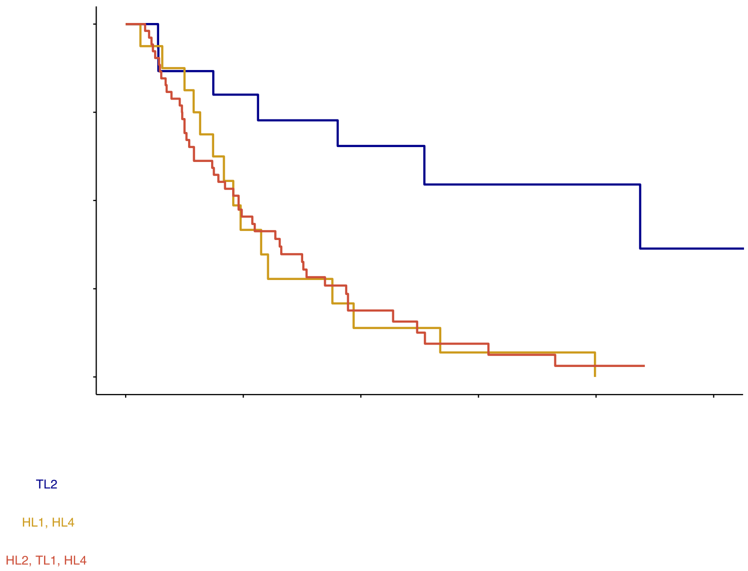

B)

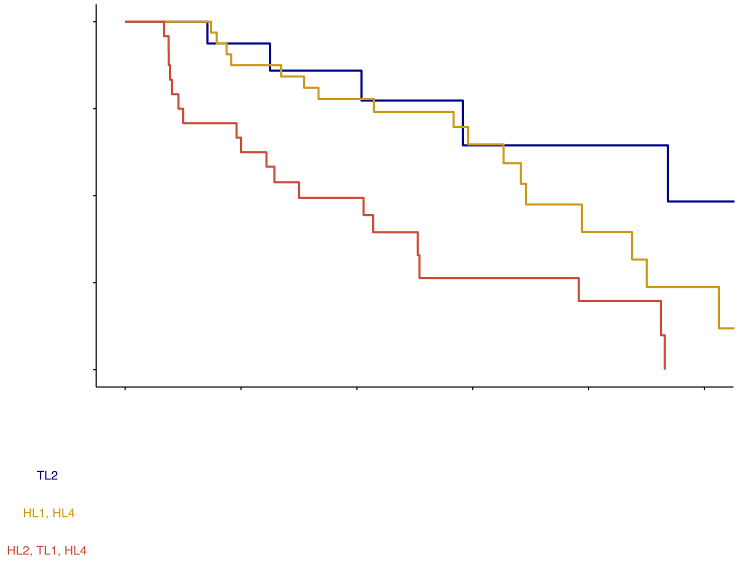

Supplementary Figure 6: RNA-SeQC quality control metrics for NGS RNA samples A) general metrics with filtered samples (n=13) for a cutoff of 0.8 median exon CV. B) PCA of filtered transcriptome colored by 3' Strand Bias C) Median Exon CV

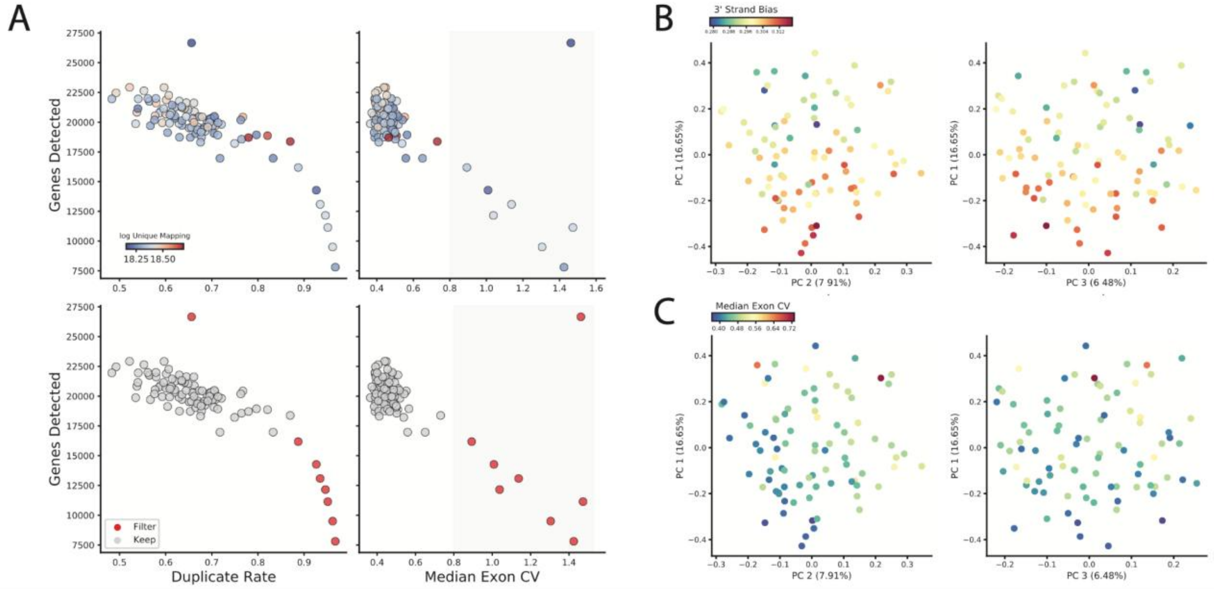

Supplementary Figure 7: Down sampling analysis of dataset for binary matrix factorization; A-D) random down-sample runs (n=100) for a range of sample sizes (n=25-214) showing explained variance (A) K-L divergence (B) Residuals (C) and Silhouette Score (D); E) Heatmap of mean scores for each down-sample run.

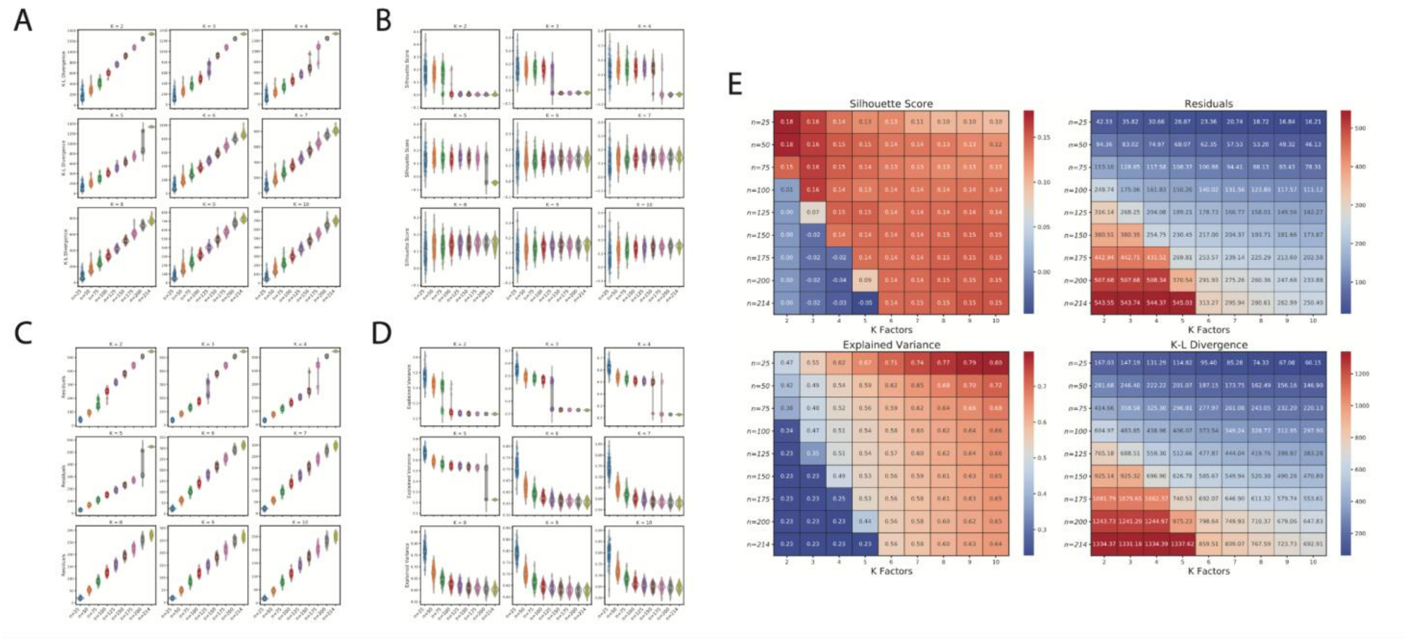

Supplement: Supplementary file 1 — Supplementary Information [file 41467_2022_30694_MOESM1_ESM.pdf]
