# Supplementary material for: Genetic subtypes of smoldering multiple myeloma are associated with distinct pathogenic phenotypes and clinical outcomes
Source: Nat Commun. 2022 Jun 15;13:3449. doi: 10.1038/s41467-022-30694-w (PMC9200804; doi:10.1038/s41467-022-30694-w)
Supplement: Supplementary file 3 — Reporting Summary [file 41467_2022_30694_MOESM3_ESM.pdf]

nature research

Corresponding author(s): Gad Getz, Irene Ghobrial

Last updated by author(s): 05/27/2021

Reporting Summary

Nature Research wishes to improve the reproducibility of the work that we publish. This form provides structure for consistency and transparency in reporting. For further information on Nature Research policies, see our Editorial Policies and the Editorial Policy Checklist.

Statistics

For all statistical analyses, confirm that the following items are present in the figure legend, table legend, main text, or Methods section.

n/a	Confirmed	
☐	☒	The exact sample size (*n*) for each experimental group/condition, given as a discrete number and unit of measurement
☐	☒	A statement on whether measurements were taken from distinct samples or whether the same sample was measured repeatedly
☐	☒	The statistical test(s) used AND whether they are one- or two-sided<br>*Only common tests should be described solely by name; describe more complex techniques in the Methods section.*
☐	☒	A description of all covariates tested
☐	☒	A description of any assumptions or corrections, such as tests of normality and adjustment for multiple comparisons
☐	☒	A full description of the statistical parameters including central tendency (e.g. means) or other basic estimates (e.g. regression coefficient) AND variation (e.g. standard deviation) or associated estimates of uncertainty (e.g. confidence intervals)
☐	☒	For null hypothesis testing, the test statistic (e.g. *F*, *t*, *r*) with confidence intervals, effect sizes, degrees of freedom and *P* value noted<br>*Give P values as exact values whenever suitable.*
☒	☐	For Bayesian analysis, information on the choice of priors and Markov chain Monte Carlo settings
☒	☐	For hierarchical and complex designs, identification of the appropriate level for tests and full reporting of outcomes
☐	☒	Estimates of effect sizes (e.g. Cohen's *d*, Pearson's *r*), indicating how they were calculated

Our web collection on statistics for biologists contains articles on many of the points above.

Software and code

Policy information about availability of computer code

Data collection	Data was collected and stored using MS Excel v16, R version 3.6.0 with the knitr v1.15.1 package for reproducible research.
Data analysis	For the Genomic analysis of the DNA and RNA sequencing data, we have utilized the Broad Institute and the Getz Lab CGA WES Characterization pipeline [https://github.com/broadinstitute/CGA_Production_Analysis_Pipeline] developed at the Broad Institute to call, filter, and annotate somatic mutations and copy number variation. Detailed descriptions of our analytical pipelines are provided in Online Methods and Supplemental Information, listing for each step the version, algorithm and parameters used. The pipeline employs the following tools:<br>FireCloud<br>BWA v0.5.9<br>ContEst Queue v1.4-437-g6b8a9e1<br>Coverage/Depth tool Firehose task GlobalCoverageByZone v23<br>TN swap tool Firehose task CrosscheckLaneFingerprintsPipeline v16<br>MuTect2 v3.6-97-g881c5e9<br>Indellocator Firehose task CallIndelsPipeline v77<br>ReCapSeg Firehose tasks ReCapSegCoverage v20 and recapseg_tumor_pcov v34<br>AllelicCapseq Firehose task AllelicCapseg v22<br>OxoG Filter Firehose task oxoGFilter_v3 v62<br>PoN filtering FireCloud task maf_pon_filter v23<br>ABSOLUTE v1.5<br>Logodds Tumor-only filter Firehose task Filter_For_Tumor_Only_Samples v10<br>deTin FireCloud tasks TumorInNormalEst v85 and deTiN_allele_shift v43

Signature Analyzer v1.1
NMF consensus clustering custom script uploaded to GitHub
MutSig 2CV v2CV
GISTIC2.0 v2.0
IGV v2.4.9
For the RNA sequencing output, we computationally processed the samples using the GTEx V8 pipeline and aligned them to Hg19 Gencode v19.
Statistical analyses for correlation with clinical variables and outcomes were performed using R version 3.6.0 (2019-04-26)
R-Studio Version 1.0.153
R v3.6.0 with these packages:
survival v2.41-2
qvalue v2.6.0
knitr v1.15.1
ggplot2 v2.2.1
fgsea v1.12.0
limma v3.42.2
ggpubr v0.4.0
EnhancedVolcano v1.4.0
Python v3.7.7
All code to reproduce results in this paper is available here: https://github.com/getzlab/SMM_clustering_2020

For manuscripts utilizing custom algorithms or software that are central to the research but not yet described in published literature, software must be made available to editors and reviewers. We strongly encourage code deposition in a community repository (e.g. GitHub). See the Nature Research guidelines for submitting code & software for further information.

Data

Policy information about availability of data

All manuscripts must include a data availability statement. This statement should provide the following information, where applicable:

- Accession codes, unique identifiers, or web links for publicly available datasets
- A list of figures that have associated raw data
- A description of any restrictions on data availability

Data Availability. The Genomic and transcriptomic data of the primary cohort generated in this study including the whole exome, targeted capture and RNA sequencing data) have been deposited in the dbGAP database under accession number phs001323.v3.p1 (https://ncbi.nlm.nih.gov/projects/gap/cgi-bin/study.cgi?study_id=phs001323.v3.p1). Access to the raw data can be obtained upon request. The other published data used as validations cohorts in this study are already deposited in public databases. For the first validation cohort11, the targeted panel data are deposited in the European Genome-phenome Archive (EGA) database under accession code EGAD00001005056 (https://ega-archive.org/datasets/EGAD00001005056). The whole-exome sequencing is deposited in the EGA database under accession code EGAD00001005285 (https://ega-archive.org/datasets/EGAD00001005285). These data are available under restricted access; access can be obtained upon request. The raw data of the published second validation cohort is deposited in the NCBI Sequence Read Archive (SRA) BioProject under accession number PRJNA541307 (https://www.ncbi.nlm.nih.gov/sra/?term=PRJNA541307)12. The remaining data are available within the Article or Supplementary Information file.

Field-specific reporting

Please select the one below that is the best fit for your research. If you are not sure, read the appropriate sections before making your selection.

- [x] Life sciences
- [ ] Behavioural & social sciences
- [ ] Ecological, evolutionary & environmental sciences

For a reference copy of the document with all sections, see nature.com/documents/nr-reporting-summary-flat.pdf

Life sciences study design

All studies must disclose on these points even when the disclosure is negative.

Sample size

1) Composition and description of the cohort: Supplemental Online Methods.
2) With 214 tumors and the identified background mutation rate, we have >98% power to detect candidate cancer genes (CCGs) in at least 10% of patients (http://www.tumorportal.org/power).
3) For the clustering analysis, minimum sample size was calculated. To select the number of clusters (K) for the consensus clustering, we randomly downsampled our input matrix and computed silhouette scores using Dice dissimilarity, residuals of factorization fit, variance explained, and K-L divergence on binary matrix factorizations over a range of K. We found a decrease in K-L divergence with our full dataset from K = 5 to K = 6, which suggested that 6 clusters were best suited to ensure a converged factorization for N = 214. Additionally, we found that variance explained stabilized when we performed down sampling analyses at N = 75-100, suggesting we were powered to perform binary matrix factorization for a cohort at this minimum size. We concluded that a minimum of 100 samples and 6 clusters were suited for this approach.

Data exclusions

Samples from patients who presented at diagnosis with overt multiple Myeloma symptoms, including hypercalcemia, renal impairment, anemia, or bone lytic lesions (CRAB), or had any myeloma-defining event were excluded from the analysis. For the analysis of association of the genetic clusters with clinical outcomes, samples from patients who enrolled in clinical trials for treatment of smoldering multiple myeloma were excluded from this analysis. The final sample size for this sub-analysis was 87 samples.

Replication	Replication of the clinical outcomes of the genetic clusters was performed through external validation in two SMM cohorts.
Randomization	This is a retrospective cohort study. Randomization was not required. The patient samples identified based on clinical state and the diagnosis of smoldering multiple myeloma. Outcomes in terms of progression to active symptomatic myeloma were assessed based on the clinical course for each patient.
Blinding	Detection of genetic clusters was performed independent of and blinded to clinical endpoints.

Reporting for specific materials, systems and methods

We require information from authors about some types of materials, experimental systems and methods used in many studies. Here, indicate whether each material, system or method listed is relevant to your study. If you are not sure if a list item applies to your research, read the appropriate section before selecting a response.

Materials & experimental systems

n/a	Involved in the study	
☒	☐	Antibodies
☒	☐	Eukaryotic cell lines
☒	☐	Palaeontology and archaeology
☒	☐	Animals and other organisms
☐	☒	Human research participants
☒	☐	Clinical data
☒	☐	Dual use research of concern

Methods

n/a	Involved in the study	
☒	☐	ChIP-seq
☒	☐	Flow cytometry
☒	☐	MRI-based neuroimaging

Human research participants

Policy information about studies involving human research participants

Population characteristics	This study used tissue specimens that have already been collected as part of a participant's clinical care. We studied a total of 214 patients with SMM at the time of diagnosis. We obtained 120 samples from Dana-Farber Cancer Institute, USA, University College London, UK, and University of Athens, Greece. In addition, we processed whole exome and RNA sequencing raw data from 94 available patient samples from a multicenter clinical trial (NCT02316106). Patient samples were collected in the period between 2007-2018. Last date of data collection of clinical outcomes for the 120 patients was October 2020. Patients who enrolled on clinical trials were removed from the analysis of the natural course of disease progression as described in details in the supplemental methods.
Recruitment	This is a multi-institutional observational study of Precursor conditions of Multiple Myeloma to assess the relationship between molecular events and progression to symptomatic overt Multiple Myeloma with identifier NCT02269592. The purpose of this research study is to perform these molecular analyses on tissues (obtained from biopsies), blood, or other body fluids such as saliva.
Ethics oversight	This study was approved by the institutional review board (IRB) of the Dana-Farber Cancer Institute and the IRBs and ethics committees of all the participating institutions. All relevant ethical regulations were followed. Informed consent was obtained from the human subjects on clinical trial. All samples were obtained after written informed consent, according to the Declaration of Helsinki.

Note that full information on the approval of the study protocol must also be provided in the manuscript.
